# Evaluation of targeting c-Src by the RGT-containing peptide as a novel antithrombotic strategy

**DOI:** 10.1186/s13045-015-0159-8

**Published:** 2015-05-30

**Authors:** Jiansong Huang, Xiaofeng Shi, Wenda Xi, Ping Liu, Zhangbiao Long, Xiaodong Xi

**Affiliations:** State Key Laboratory of Medical Genomics, Shanghai Institute of Hematology, Collaborative Innovation Center of Hematology, Ruijin Hospital, Shanghai Jiao Tong University School of Medicine, 197 Second Ruijin Road, Shanghai, 200025 China; Shanghai Institute of Hypertension, Ruijin Hospital, Shanghai Jiao Tong University School of Medicine, 197 Second Ruijin Road, Shanghai, 200025 China; Sino-French Research Center for Life Sciences and Genomics, Ruijin Hospital, Shanghai Jiao Tong University School of Medicine, 197 Second Ruijin Road, Shanghai, 200025 China

**Keywords:** Antithrombotic agent, Blood platelets, Drug design, Integrin αIIbβ3, c-Src

## Abstract

**Background:**

Interaction of integrin β3 with c-Src plays critical roles in cellular signaling which is heavily implicated in platelet adhesion and aggregation, as well as in tumor cell proliferation and metastasis or in osteoclastic bone resorption. Selectively blocking integrin αIIbβ3 outside-in signaling in platelets has been a focus of attention because of its effective antithrombotic potential together with a sufficient hemostatic capacity. The myristoylated RGT peptide has been shown to achieve this blockade by targeting the association of c-Src with the integrin β3 tail, but the lack of key information regarding the mechanisms of action prevents this strategy from being further developed into practical antithrombotics. Therefore, in-depth knowledge of the precise mechanisms for RGT peptide in regulating platelet function is needed to establish the basis for a potential antithrombotic therapy by targeting c-Src.

**Methods:**

The reduction-sensitive peptides were applied to rule out the membrane anchorage after cytoplasmic delivery. The c-Src activity was assayed at living cell or at protein levels to assess the direct effect of RGT targeting on c-Src. Thrombus formation under flow in the presence of cytoplasmic RGT peptide was observed by perfusing whole blood through the collagen-coated micro-chamber.

**Results:**

The RGT peptide did not depend on the membrane anchorage to inhibit outside-in signaling in platelets. The myr-AC ~ CRGT peptide readily blocked agonist-induced c-Src activation by disrupting the Src/β3 association and inhibited the RhoA activation and collagen-induced platelet aggregation in addition to the typical outside-in signaling events. The myr-AC ~ CRGT had no direct effect on the kinase activity of c-Src in living cells as evidenced by its inability to dissociate Csk from c-Src or to alter the phosphorylation level of c-Src Y^416^ and Y^527^, consistent results were also from in vitro kinase assays. Under flow conditions, the myr-AC ~ CRGT peptide caused an inhibition of platelet thrombus formation predominantly at high shear rates.

**Conclusions:**

These findings provide novel insights into the molecular mechanisms by which the RGT peptide regulates integrin signaling and platelet function and reinforce the potential of the RGT peptide-induced disruption of Src/β3 association as a druggable target that would finally enable in vivo and clinical studies using the structure-based small molecular mimetics.

**Electronic supplementary material:**

The online version of this article (doi:10.1186/s13045-015-0159-8) contains supplementary material, which is available to authorized users.

## Background

Arterial thrombosis developing on the atherosclerotic lesions causes heart attack and stroke that are currently the most common cause of death. Platelets play central roles in hemostasis and in arterial thrombosis by forming thrombi [1]. Considerable efforts in developing antiplatelet or antithrombotic drugs have been directed towards integrin αIIbβ3 since it is exclusively expressed in the platelet/megakaryocyte lineage and serves as a final common pathway of platelet aggregation in response to various agonists.

Arterial thrombus formation is initiated by platelet adhering to the injured vessel wall at high shear rates via the interaction of glycoprotein Ib (GPIb) with von Willebrand factor (vWF) [2]. The adherent platelets need to be further activated primarily mediated by integrin αIIbβ3 so as to make firm association (spreading) and to recruit more platelets (aggregation). The αIIbβ3 activation is tightly regulated by inside-out signals initiated by various receptor/ligand interactions such as glycoprotein VI (GPVI)/collagen, P2Y_1_ or P2Y_12_/adenosine diphosphate (ADP) and protease-activated receptor (PAR)/thrombin, etc. The transduction of inside-out signals transforms the integrin αIIbβ3 receptor from low to high affinity that facilitates ligand (fibrinogen) binding and thus platelet aggregation [3]. Ligand binding to integrin αIIbβ3 triggers outside-in signaling that promotes platelet cytoskeletal reorganization leading to the firm association of platelets with the vessel wall or with each other. Integrin αIIbβ3 bidirectional signaling is primarily dependent on the complex and dynamic associations between the cytoplasmic proteins and the short cytoplasmic tail of integrin β3. For instance, talin and kindlin bind to the β3 NxxY motifs and play central roles in inside-out signaling [4], and the association of c-Src with the β3 tail, the RGT residues in particular [5, 6], is obligatory for outside-in signaling [5]. The conventional antagonists prevent ligand binding to αIIbβ3 receptor and thus actually inhibit the platelet functions regulated by both inside-out and outside-in signals.

Genetically manipulated mouse models with an impaired integrin αIIbβ3 outside-in signaling [3, 7–14] showed a significantly compromised potential of arterial thrombosis with a normal or only slightly prolonged bleeding time, indicating that selective inhibition of outside-in signaling may represent a rational antithrombotic strategy. Compared with the genetic manipulation, which seems currently impossible to be applied as a therapy, synthetic molecules with “drug-like” properties have the capability of being developed into drugs. We have previously established that disruption of the Src/β3 interaction by the myristoylated RGT peptide selectively inhibited outside-in signaling [15]. A recent study also showed that the myr-FEEERA peptide disrupted Gα13/β3 interaction, ultimately hampering c-Src activation and thereby inhibiting outside-in signaling [16]. These data, in light of the established mechanisms in which c-Src is pivotal for outside-in signaling by interacting with β3 [5, 17, 18], suggest that the Src/β3 association may be a potential target for the development of antithrombotics that do not cause excessive bleeding. The synthesized molecules, such as the RGT peptide, may thus serve as a paradigm for the conception of novel antithrombotic therapies even though the peptide itself may unlikely be directly applied as a drug.

The myristoylation modification renders the peptide membrane permeable but concomitantly integrates the peptide into the cell membrane that may enhance its ability to compete with β3 for c-Src [19]. It thus became essential to define whether the RGT peptide relies on the membrane anchorage to exert its effects. This important feature is a very basic prerequisite for designing further applications of the peptide or its analogues. Activated c-Src is implicated in a wide range of cellular functions, and there is evidence that binding of some peptides to different domains of c-Src has direct impacts on its kinase activity most likely because of the conformational regulations. For example, Nef binding to the Src homology 3 (SH3) domain or pYEEI to Src homology 2 (SH2) [20] induced an increased activity of Src-family kinases, as well as RGT peptide binding to SH3 domain of c-Src primed the kinase [21]. Before moving towards the in vivo and clinical studies using the structure-based small molecular mimetics, the consequences of the RGT peptide, or its analogues, binding to c-Src in the context of its kinase activity need to be carefully defined. A vast body of experimental data, mostly in vivo, indicated that outside-in signaling was essential to stable platelet adhesion and aggregation [8, 9, 16], and impaired stabilization of the developing thrombi under flow conditions might most likely be ascribable to the disruption of outside-in signaling [22, 23]. The RGT peptide has been reported to selectively inhibit outside-in signaling under static condition [15], whether and if so, how it influences thrombus formation under flow condition is yet an important but unanswered question.

By using reduction-sensitive peptides, the present study aimed to address whether the RGT peptide depends on membrane anchorage to influence Src-regulated signaling and whether it alters the kinase activity of c-Src. In addition, the effect of selective blockade of outside-in signaling in human platelets by the RGT peptide on thrombus formation and growth under flow was tested. These efforts allow evaluating the potential of the intracellular delivery of the RGT analogues for antithrombotic therapies where an effective inhibition on thrombosis is accomplished together with an adequate hemostasis.

## Results

### A thorough reduction of myr-AC ~ CRGT peptide is achieved in platelet cytoplasm

**Fig. 1:**
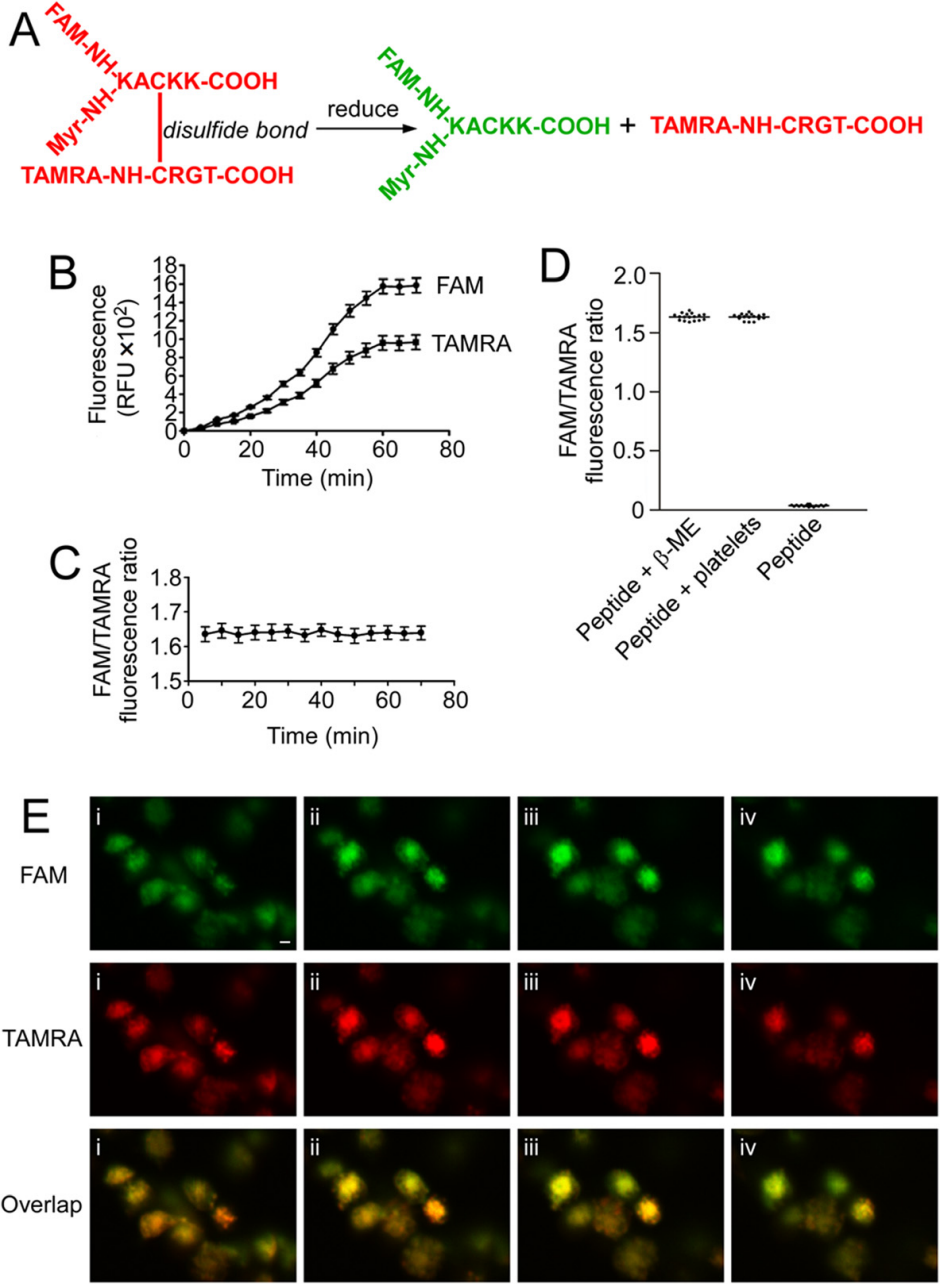
Membrane-permeable and reduction-sensitive peptide produced free CRGT peptide upon entering platelet cytosol. **a** Schematic presentation shows that the reduction of disulfide bonds led to a separation of the FAM fluorescence donor and the TAMRA fluorescence acceptor that dequenched the fluorescence of FAM allowing detection by a spectrofluorometer. **b** Washed platelets were treated with the reduction-sensitive fluorescent peptide. The fluorescence at 576 nm (TAMRA) and 519 nm (FAM) increased over time. **c** The ratio of FAM/TAMRA fluorescence quantum was calculated and presented as the mean and SD of three independent experiments. **d** Comparison of the FAM to TAMRA fluorescence ratio among peptide-treated platelets (peptide + platelets), 0.3 M β-ME-treated peptide (peptide + β-ME), or untreated peptide (peptide). The data were measured and presented as same as in panel **c. e** Confocal analysis of the peptide-treated platelets were performed utilizing Z-stack scanning with intervals of 1.2 μm (from *i* to *iv*). Images are from a single experiment representative of three so performed, scale bar represents 2 μm

It has been established that myristoylated RGT peptide selectively inhibited integrin αIIbβ3-mediated outside-in signaling [15]. In order to define the underlying mechanisms with respect to membrane anchorage, a cell membrane-permeable and reduction-sensitive fluorescent peptide (Fig. 1a) was employed in this work. Since the emission spectrum of FAM overlaps the excitation spectrum of TAMRA, the physical proximity of FAM to TAMRA is able to quench the green fluorescence of FAM [24]. Platelets were incubated with the peptide and the fluorescence intensity of the TAMRA and FAM in the platelets was measured, and both were found increased over time and reached a plateau phase at 60 min (Fig. 1b). We noticed a proportional increase of two fluorescent signals indicating a disulfide bond cleavage upon entering the cytoplasm. The ratio of FAM to TAMRA fluorescence quantum was therefore measured and showed no significant difference at different incubation time (Fig. 1c) suggesting a quick cleavage of the disulfide bond, which was attributed to a strong reductive potential in platelet cytosol similar to the effect of 0.3 M of β-mercaptoethanol (β-ME) (Fig. 1d). Such a vigorous reduction of disulfide bond of the internalized peptide in platelet cytosol precludes a scenario in which the membrane-integrated myr-AC ~ CRGT is able to retain its CRGT moiety so as to associate with the membrane. Confocal microscopic results from platelets treated for 60 min also showed that both green FAM and red TAMRA existed homogeneously in the cytoplasm (Fig. 1e), indicating a dynamic balance of the myristoylation moiety between staying membrane-bound and in cytoplasm as shown in previous reports [15, 25, 26].

### Activated c-Src directly phosphorylates β3 Y^747^ and Y^759^ independent of its binding to the RGT sequence of the β3 tail

**Fig. 2:**
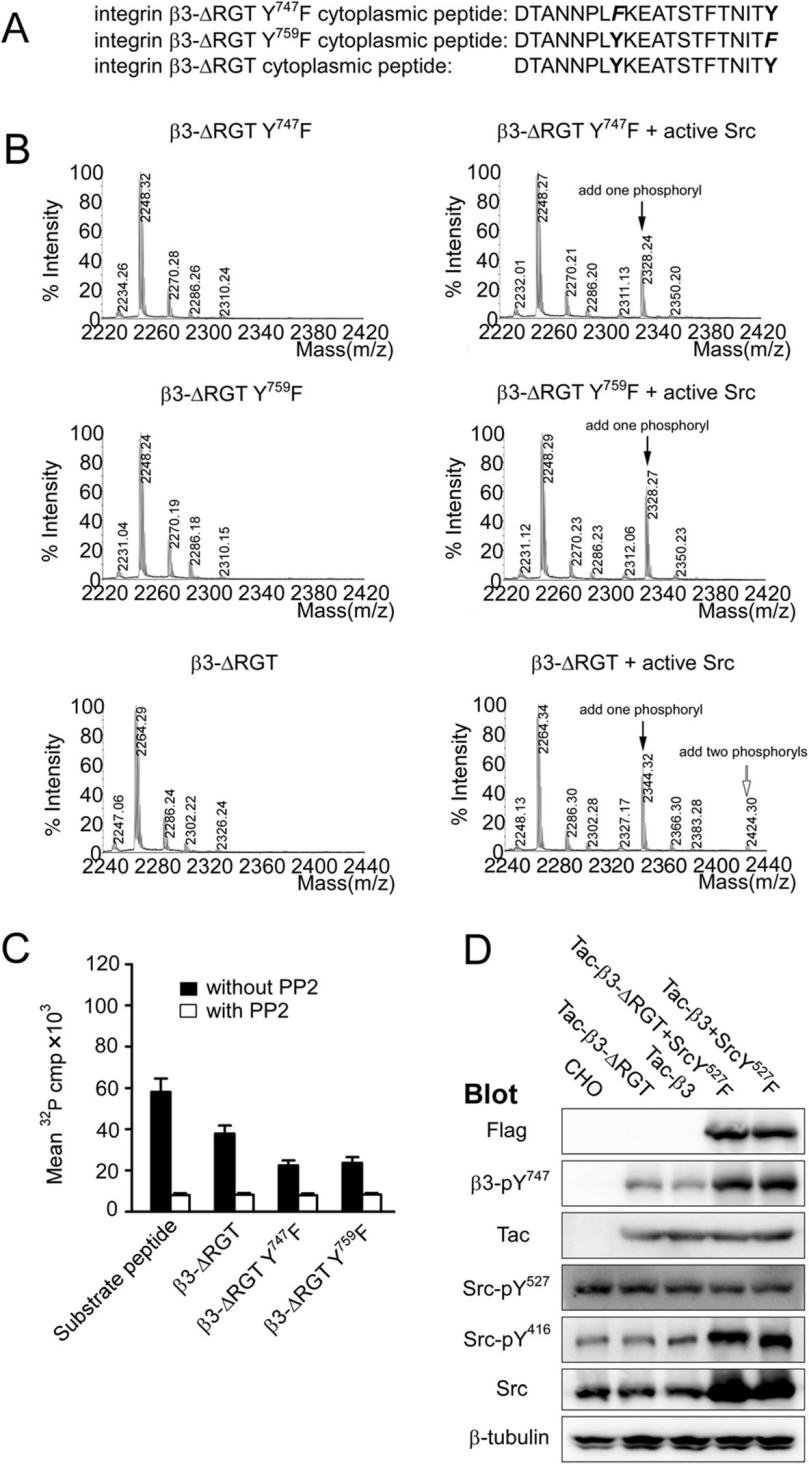
The RGT sequence of the β3 tail was not necessary for the direct phosphorylation of the β3 Y^747^ and Y^759^ by active c-Src. **a** The amino acid sequences for the integrin β3-ΔRGT cytoplasmic peptides, or with Y^759^F, or Y^747^F mutations. **b** MALDI-TOF-MS results of the differently mutated β3 cytoplasmic peptides incubated with or without active c-Src. *Black* and *blank* arrows denote the new peak with one and two phosphorylated tyrosine(s), respectively. **c** [γ-^32^P]ATP incorporation of differently mutated β3 cytoplasmic peptides by active c-Src. Results are presented as mean and SD of three independent experiments. **d** Western blot analysis of the tyrosine phosphorylation of Tac-β3-ΔRGT. The phosphorylation level of Y^747^ residue in CHO cells expressing Tac-β3-ΔRGT or Tac-β3 together with activated c-Src was similar, but stronger than in those expressing only Tac-β3-ΔRGT or Tac-β3

**Fig. 3:**
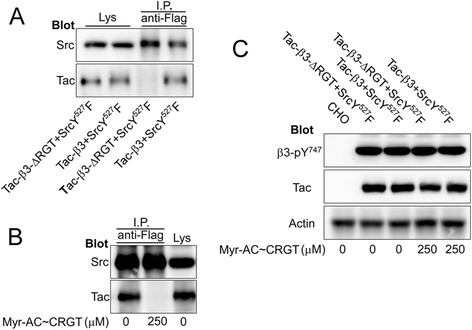
Disruption of the constitutive Src-β3 association did not affect the tyrosine phosphorylation of the β3 tail by active c-Src. **a** Lysates of the CHO cells expressing both Tac-β3 (or Tac-β3-ΔRGT) and c-Src Y^527^F were immunoprecipitated with anti-Flag antibody (for c-Src Y^527^F) and the immune complexes were subjected to SDS-PAGE and blotted with an anti-Tac monoclonal antibody. Tac-β3 but not Tac-β3-ΔRGT was co-immunoprecipitated with c-Src Y^527^F. **b** Lysates of CHO cells co-expressing Tac-β3 and c-Src Y^527^F pretreated with 250 μM of myr-AC ~ CRGT were immunoprecipitated by an anti-Flag antibody. The association of Src Y^527^F with β3 was disrupted by myr-AC ~ CRGT peptide. **c** Lysates of CHO cells co-expressing Tac-β3 (or Tac-β3-ΔRGT) and c-Src Y^527^F pretreated with 250 μM of myr-AC ~ CRGT were analyzed by Western blot using anti-β3-pY^747^ and anti-Tac antibodies. Myr-AC ~ CRGT did not affect the tyrosine phosphorylation of the β3 tail no matter whether it contains the RGT sequences. Actin served as a loading control

The RGT sequence of the β3 tail mediated a direct interaction with the SH3 domain of c-Src that is critical for outside-in signaling [5] and the activated c-Src phosphorylated the tyrosines of the integrin β3 tail [27] that were confirmed by our current observations (Additional file 1: Figure S1). We thus asked whether the kinase requires binding to the β3 RGT sequence to achieve this phosphorylation action. The mutated β3 cytoplasmic peptides devoid of the RGT sequence (Fig. 2a) were employed. The mass spectrometry results (Fig. 2b) and [γ-^32^P] adenosine triphosphate (ATP) incorporation assays (Fig. 2c) showed that the tyrosine residue(s) could be unequivocally phosphorylated. This catalytic process was further confirmed in living Chinese hamster ovary (CHO) cells bearing Tac-β3-ΔRGT chimeras and c-Src Y^527^F (Fig. 2d). Co-immunoprecipitation data demonstrated that the c-Src Y^527^F formed a complex with Tac-β3 but not with Tac-β3-ΔRGT (Fig. 3a). The CHO cells expressing c-Src Y^527^F and Tac-β3 were treated with myr-AC ~ CRGT peptide and results showed that the disruption of the c-Src Y^527^F interaction with β3 by myr-AC ~ CRGT (Fig. 3b) did not attenuate the phosphorylation of the Y^747^ (Fig. 3c).

### Myr-AC ~ CRGT peptide *per se* does not directly alter the activity of c-Src

**Fig. 4:**
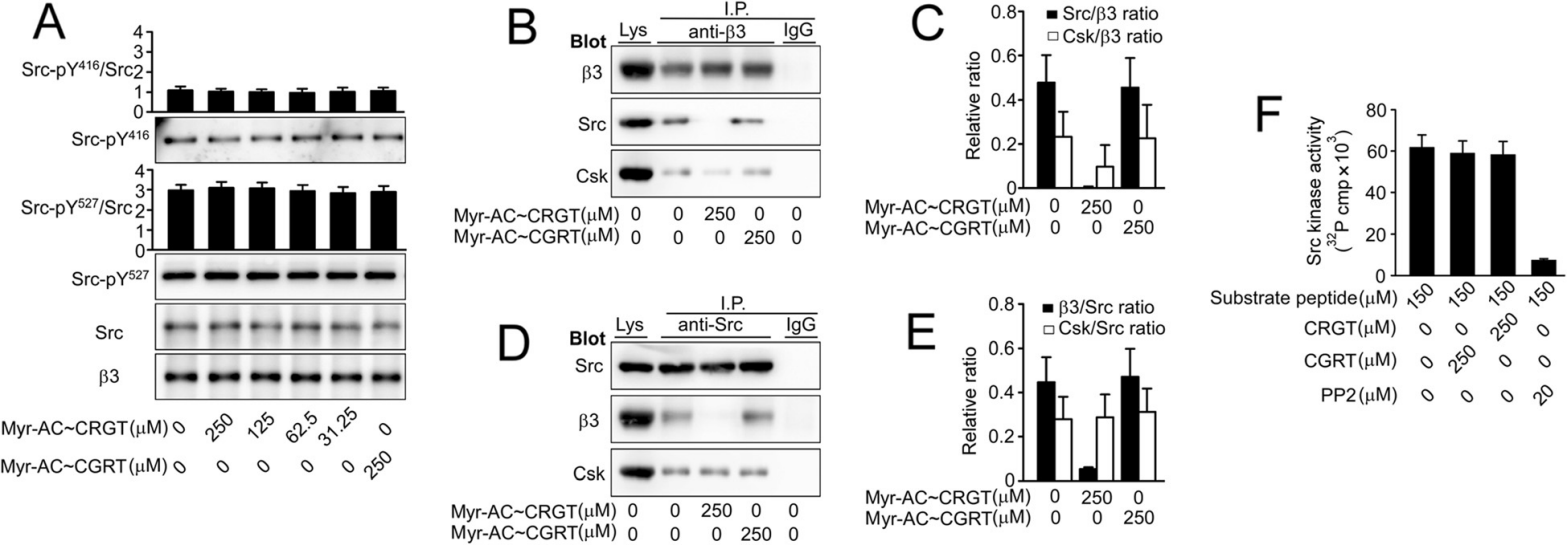
Myr-AC ~ CRGT peptide per se did not directly alter the activity of c-Src. **a** Effect of myr-AC ~ CRGT on c-Src activity in platelets. The peptide-pretreated platelets were lysed and blotted for Src-pY^416^, Src-pY^527^, Src or β3. The band intensity was quantified using the NIH Image J software. The extent of Src Y^416^ or Y^527^phosphorylation was expressed as a ratio of Src-pY^416^ or Src-pY^527^ signals versus total Src signals. Integrin β3 served as a loading control. **b** Effect of myr-AC ~ CRGT on the interaction of integrin β3 with c-Src or Csk. The peptide-pretreated platelets were lysed and immunoprecipitated with anti-β3 antibody and then analyzed with anti-β3, anti-Src, and anti-Csk antibodies. **c** Densitometric presentations of the results in panel **b**. **d** Effect of myr-AC ~ CRGT on the interaction of c-Src with integrin β3 or Csk. **e** Densitometric presentations of the results in panel **d**. Results are shown the mean and SD of three independent experiments. **f** Substrate peptide KVEKIGEGTYGVVYK was mixed with purified active c-Src in the presence or absence of the CRGT, CGRT peptides, or PP2 and then the [γ-^32^P]ATP incorporation was measured. Data are shown as the mean and SD of three independent experiments

It is known that active c-Src is implicated in a wide range of cellular functions and that Nef binding to the SH3 domain or pYEEI to SH2 [20] induces an increased activity of Src-family kinases, as well as RGT peptide binding to SH3 domain of c-Src primes the kinase [21]. Given that the RGT peptide competes with β3 for c-Src by binding to the SH3 domain, the consequences of RGT peptide binding to c-Src with respect to its kinase activity need to be clarified. Platelets were incubated with different concentrations of myr-AC ~ CRGT and assayed for c-Src activity. There was no difference of the c-Src Y^416^ and Y^527^ phosphorylation level between myr-AC ~ CRGT-treated and control samples (Fig. 4a). Co-immunoprecipitation with anti-β3 antibody showed that myr-AC ~ CRGT abolished the interaction of β3 with c-Src and remarkably inhibited that with Csk (Fig. 4b and c), while data with anti-Src antibody exhibited a strong disruption of interaction of Src/β3 but not that of Src/Csk by myr-AC ~ CRGT (Fig. 4d and e). We further examined the effect of CRGT peptide on the activity of c-Src with a Src kinase assay kit. In contrast to a significant inhibition caused by PP2, CRGT did not alter the kinase activity of c-Src (Fig. 4f).

### Myr-AC ~ CRGT inhibits agonist-induced c-Src activation and the phosphorylation of the β3 cytoplasmic tyrosines via separating c-Src from the β3 tail

**Fig. 5:**
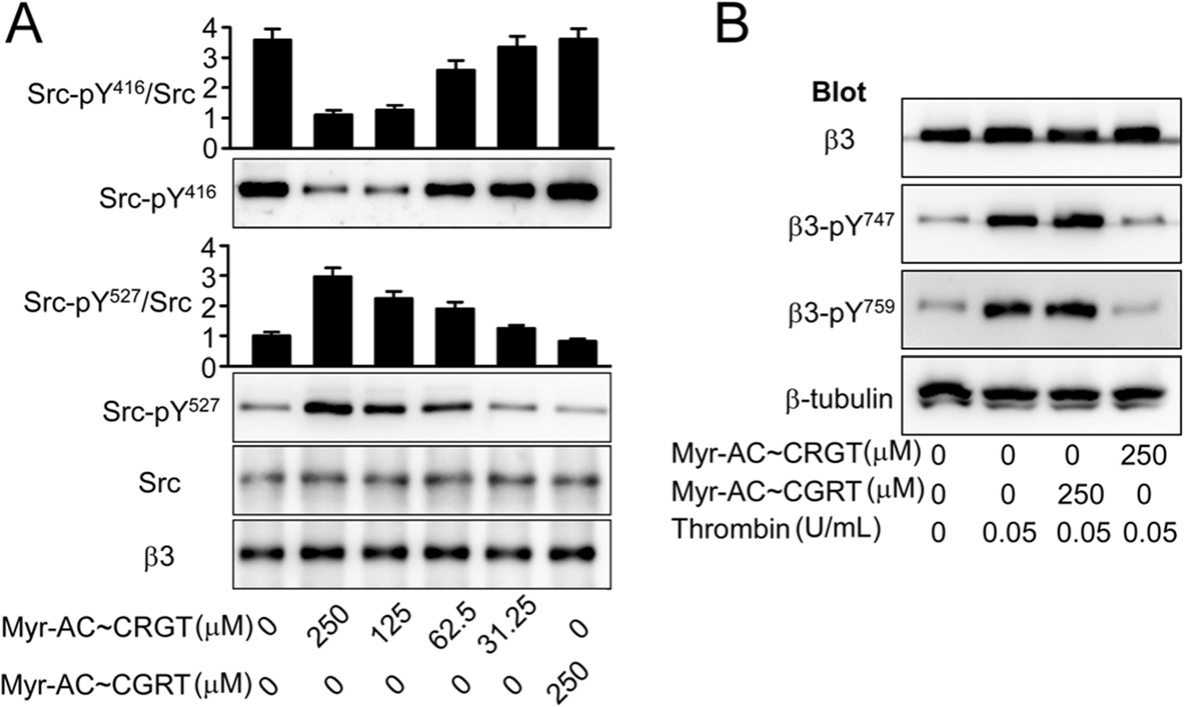
Myr-AC ~ CRGT inhibited the phosphorylation of the integrin β3 cytoplasmic tyrosines in thrombin-stimulated platelets. **a** Effect of myr-AC ~ CRGT on c-Src activity in platelets in response to thrombin stimulation. After an incubation with myr-AC~CRGT or myr-AC~CGRT, platelets were stimulated by thrombin then lysed and blotted with anti-Src-pY^416^, anti-Src-pY^527^, and anti-Src antibodies. Integrin β3 served as a loading control. Results are shown as the mean and SD of three independent experiments. **b** Effect of myr-AC ~ CRGT on thrombin-induced phosphorylation of the Y^747^ and Y^759^ of the β3 cytoplasmic tail. After an incubation with 250 μM of myr-AC ~ CRGT or myr-AC ~ CGRT, platelets were stimulated by thrombin (0.05 U/mL) with stirring, then were lysed in SDS-PAGE sample buffer and analyzed by Western blot using anti-β3, anti-β3-pY^747^, anti-β3-pY^759^, or anti-β-tubulin antibodies. Untreated platelets were used as a negative control, thrombin-stimulated platelets as a positive control, and β-Tubulin as a loading control

Free CRGT in cytoplasm produced upon the internalization of myr-AC ~ CRGT peptide is supposed to compete with β3 for binding to c-Src and prevent the kinase from being activated and participating in outside-in signaling. Indeed, myr-AC ~ CRGT treatment resulted in a disassociation of c-Src from β3 both in platelets (Fig. 4b) and CHO cells (Fig. 3b). Data further show that in thrombin-stimulated platelets, myr-AC ~ CRGT inhibited Y^416^ phosphorylation or Y^527^ dephosphorylation of c-Src in a dose-dependent manner (Fig. 5a). In platelets, myr-AC ~ CRGT (250 μM) almost completely inhibited the thrombin-induced c-Src activation; thus, the phosphorylation of β3 Y^747^ and Y^759^ residues was abolished (Fig. 5b).

### Myr-AC ~ CRGT inhibits thrombus formation under high shear rates

**Fig. 6:**
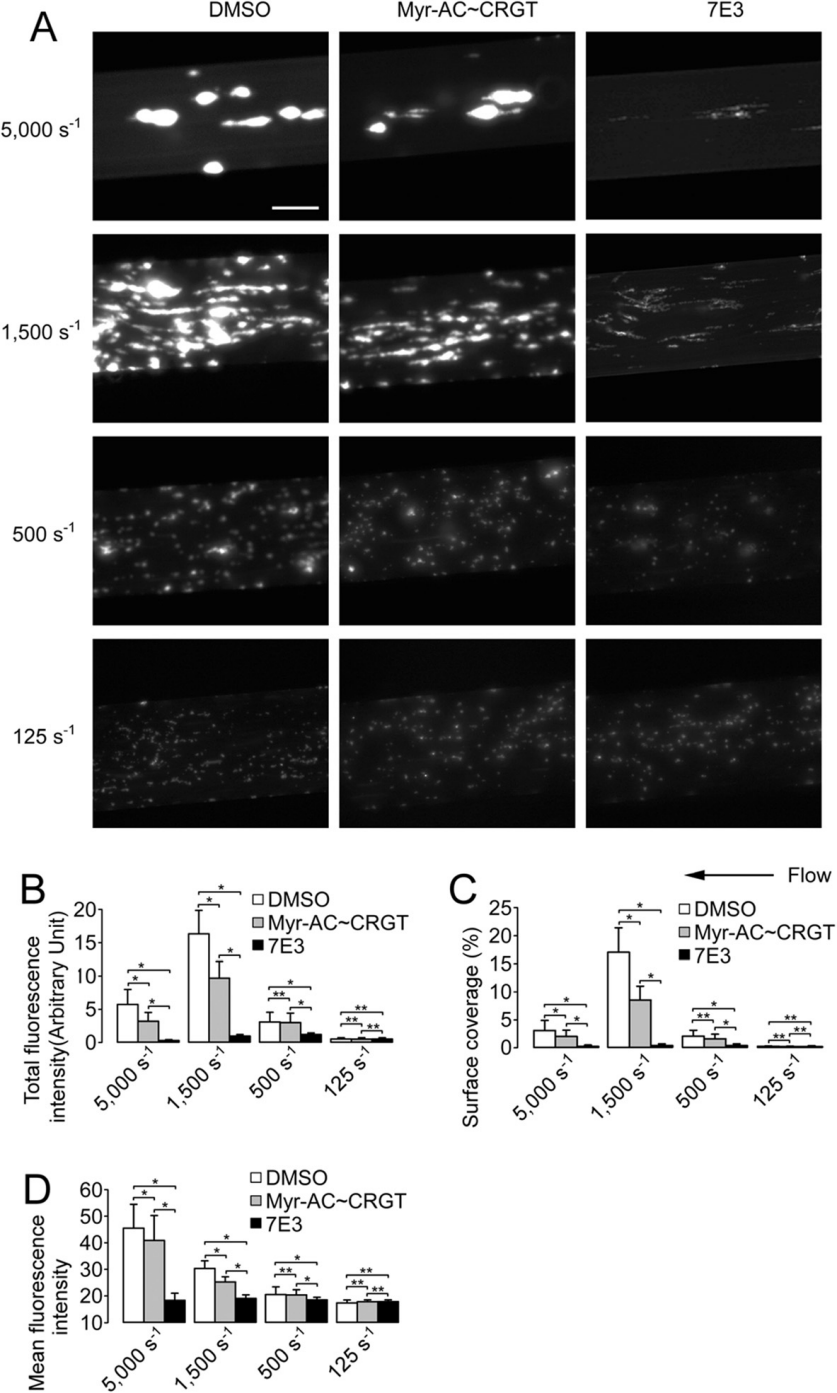
Effect of myr-AC ~ CRGT on thrombus formation of human platelets at different shear rates. Calcein AM-labeled whole blood was incubated with DMSO, myr-AC ~ CRGT (250 μM), or 7E3 prior to perfusion, and then perfused at different wall shear rates (5000, 1500, 500, or 125 s^−1^) for 4 min. The thrombus formation was observed and imaged under an inverted fluorescent microscope. **a** The representative images showed platelet thrombi (scale bar is 100 μm). **b** The quantitative data were acquired as platelet-integrated total fluorescence intensity. **c** The percentage of surface coverage. **d** The mean fluorescence intensity calibrated by surface coverage. Results are the mean and SD from three independent experiments, and 40 visual fields were randomly chosen for each experiment. **P > 0.05; *P < 0.01

The establishment of the influence of myr-AC ~ CRGT on platelet function under static conditions (Additional file 2: Figure S2 and Additional file 3: Figure S3) motivated us to examine the contribution of selectively impaired outside-in signaling to platelet adhesion and aggregation under flow conditions. The initial transient platelet adhesion and rolling under flow are mediated by the interaction of GPIb with collagen-bound vWF [28] and integrin α2β1, or GPVI binding to collagen may also contribute to this platelet/subendothelium interaction [29]. These events mediate platelet stable adhesion and irreversible aggregation primarily through integrin αIIbβ3 outside-in signaling [30]. At a wall shear rate of 1500 s^−1^, myr-AC ~ CRGT dose-dependently inhibited thrombus formation up to 250 μM (Additional file 4: Figure S4). Then, the effect of 250 μM of myr-AC ~ CRGT was further tested at different shear rates. At high shear rate (5000 s^−1^ or 1500 s^−1^), which respectively represented flow conditions in stenotic arteries and, or in arterioles or slightly stenotic arteries, thrombus formation in the presence of myr-AC ~ CRGT was between that of DMSO or αIIbβ3 antagonists where thrombus formation scarcely occurred (Fig. 6). Similar to the published observations [31, 32], significantly few platelets adhered to the surfaces under lower shear rates even in DMSO control. At intermediate shear rate (500 s^−1^), mimicking medium-sized arteries, myr-AC ~ CRGT showed no inhibitory effect in contrary to a substantial inhibition by 7E3 (Fig. 6). However, at low shear rate (125 s^−1^), which modeled the flow in venules, neither myr-AC ~ CRGT nor 7E3 inhibited platelet adhesion (Fig. 6).

## Discussion

The antagonists target integrin αIIbβ3 receptor and inhibit thrombosis but compromise hemostasis as well. Targeting signaling pathways instead of receptors may reconcile this conflict. It has been established that model animals with impaired outside-in signaling feature a reduced potential of thrombosis without excessive bleeding, and the strategy of blocking outside-in signaling has thus a definite advantage in designing new antithrombotic therapies. The myr-RGT peptide is known to be capable of blocking outside-in signaling by targeting c-Src and is considered as a synthetic molecule that is closest to be developed into drugs by virtue of structure-based analogues. However, there are key issues to be addressed before proceeding with the studies. A very primary issue is whether the inhibition of outside-in signaling by myr-RGT peptide depends on its membrane anchorage since a precise knowledge of the peptide action is definitely necessary for conceiving a structural analogue, i.e. membrane associable or not. Previous studies indicated that the regulatory effect of the myristoylated peptides on the cellular biological functions depends on its anchorage to cell membrane in some reports [33, 34] whereas it seems not the case in others [25, 26]. Glutathione is abundant in platelet cytoplasm at millimolar levels [35], which are sufficient to exhaust the capacity of the disulfide bonds of myr-AC ~ CRGT peptide at its working concentrations and confer an inability of the CRGT peptide to associate with the myristoylation moiety, and thereby with the cell membrane. In other words, the competition of the CRGT peptide with the β3 tail for c-Src is unlikely attributed to the proximity of these molecules achieved by their membrane integration. Results showed that myr-AC ~ CRGT peptide penetrated the cell membrane and yielded free CRGT peptide in platelet cytoplasm (Fig. 1). This peptide did not disrupt the integrity of the cell membrane (Additional file 5: Figure S5) but did exert an inhibitory effect on platelet spreading on immobilized fibrinogen, clot retraction, irreversible aggregation, and P-selectin expression, without affecting soluble fibrinogen binding to αIIbβ3 and reversible aggregation (Additional file 2: Figure S2 and Additional file 3: Figure S3). In contrast to active c-Src phosphorylating β3 Y^747^ and Y^759^ independent of its binding to the RGT sequence of the β3 tail in vitro (Fig. 2), c-Src in platelets is constitutively associated with β3 in an inactive form, and is activated upon signaling through αIIbβ3. Once c-Src is dissociated by myr-AC ~ CRGT from β3, it will not be activated through signaling and is thus unable to phosphorylate the β3 tail. Indeed, myr-AC ~ CRGT inhibited agonist-induced c-Src activation (Fig. 5a) and attenuated the phosphorylation of the Y^747^ and Y^759^ residues of β3 in thrombin-stimulated platelets (Fig. 5b). These data brought new information, in comparison with those from myr-RGT, that the cytoplasmic existence of free RGT peptide is sufficient to sequestrate c-Src to an extent capable of separating it from β3 (Figs. 3b and 4b). This study further provides evidence that myr-AC ~ CRGT treatment did not affect the level of c-Src activation in platelets deposited on the immobilized fibrinogen (Additional file 2: Figure S2D), but did cause a downregulation of the platelet RhoA activation in clots (Additional file 2: Figure S2G) as well as a substantial inhibition on collagen-induced platelet aggregation (Additional file 2: Figure S2H). These results suggest that the RGT peptide regulated outside-in signaling by competing with β3 for c-Src not necessarily owing to its membrane association but, rather, through sequestrating c-Src and further indicated that the membrane anchorage is thus not an issue of importance in conceiving “drug-like” molecules mimicking the active structure of RGT peptide.

In line with the previous reports, the present observations showed that active c-Src directly phosphorylates β3 Y^747^ and Y^759^ independent of its binding to the RGT sequence of the β3 tail (Fig. 2) indicating that activated c-Src may still be able to promote αIIbβ3 signaling even having been separated from the β3 tail and in view of the fact that the free RGT peptide sequestrates c-Src in cytoplasm and that some peptides binding to c-Src has direct impact on its kinase activity [20, 21]. It became essential to clarify whether the RGT peptide binding is able to alter the enzymatic activity of c-Src and accordingly to change the biological processes correlating with this multi-functional kinase. Our data show that the phosphorylation levels of Y^416^ and Y^527^ as well as the association of Csk with c-Src were not affected in platelets in the presence of free cytoplasmic CRGT peptide (Fig. 4). In addition, in vitro assays for c-Src activity, in which the phosphoryl transfer reaction occurs exclusively on the Src substrate peptide since the CRGT peptide contains no tyrosine residue, also revealed that the CRGT peptide did not influence the potential of c-Src to catalyze its substrates (Fig. 4f). These results suggest that RGT peptide binding to c-Src was unable to alter the c-Src activity and the presence of the cytoplasmic CRGT peptide caused primarily the dissociation of the Src/β3 interaction without a pronounced direct effect on c-Src. This feature for the RGT peptide argues in favor of the safety of its potential application in antithrombotic therapies.

Thrombosis occurs in the blood stream, and the shear forces play significant roles throughout the process. Selective inhibition of outside-in signaling was achieved by RGT peptide in human platelets based on experiments in static conditions [15]. We thus became interested in testing the effect of myr-AC ~ CRGT on thrombus formation under flow conditions. We chose collagen to coat the micro-chambers because it is the major component of the subendothelial matrices and has thus been recommended by the Biorheology Subcommittee of the SSC of the ISTH [36]. Platelet stable adhesion and irreversible aggregation regulated primarily through outside-in signaling [30] contribute to the thrombus growth under flow. In a very recent publication [37], Stalker and colleagues proposed a model for hemostatic plug formation in which the plugs are comprised of distinct regions defined by the degree of platelet activation, packing density, and stability. Platelets in the “core” are characterized by contact-dependent signaling, P-selectin exposure with a greater packing density while those in the unstable “shell” are loosely packed and less activated most likely dependent on the P2Y_12_ receptors. Similar features may also exist in platelets that have undergone bidirectional or inside-out signaling through integrin αIIbβ3, respectively. Therefore, the RGT-treated platelets, in which only inside-out signals can be transduced, may still be able to form thrombi though less stable to resist the hydrodynamic drag forces. Indeed, at a shear rate of 125 s^−1^, neither myr-AC ~ CRGT nor 7E3 affected the platelet adhesion, in line with the previous observations in which thrombasthenic platelets adhered normally to deendothelialized vessels at low shear rates [38, 39]. The inability of myr-AC ~ CRGT to affect platelet adhesion and aggregation remained at the intermediate shear rates (500 s^−1^) in contrast to an unequivocal inhibition induced by 7E3 (Fig. 6). At high shear rates (1500 and 5000 s^−1^) myr-AC ~ CRGT partially inhibited thrombus formation, as evidenced by the smaller and thinner thrombi and less thrombus coverage as well (Fig. 6), whereas 7E3 could still vigorously inhibit platelet adhesion and aggregation leading to a drastic decrease of thrombus formation either in size or in coverage by showing only few platelets adhered on the surfaces. This was the first application of human platelets with specifically impaired outside-in signaling in such an observation indicating that the Src-regulated outside-in signaling played a pivotal role in inducing the firm linkage of platelets to the subendothelial matrices and to each other which renders the growing thrombi more resistant to the drag forces. Hemostasis usually occurs in small vessels such as arterioles, capillaries, and venules [40] where blood loss will end as long as small numbers of platelets accumulate at the sites of injury [37]. The flow experimental data allow us to infer that in venules (<500 s^−1^), myr-AC ~ CRGT-treated platelets may adhere normally, while in arterioles and capillaries (1500 s^−1^ or greater), the unaffected inside-out signaling may still enable these platelets to form thrombi to a size sufficient to seal the vessel damages in contrast to a much more profound inhibition of 7E3 [31, 41]. This unique effect of myr-AC ~ CRGT on thrombus formation under flow may explain the consequences of the elimination of outside-in signaling in hemostatic potential manifested in genetically manipulated animals. Differently, pathological thrombosis takes place in stenotic medium-sized arteries with a luminal diameter exceeding 100 μm, usually in millimeters such as coronary or cerebral arteries. Coronary thrombosis model showed that shear rates reached as high as 84,000 s^−1^ for a 65 % stenosis by diameter [42]. Occlusion of these arteries requires bigger and more stable thrombi formed under these conditions. Significantly smaller and thinner thrombi formed by myr-AC ~ CRGT-treated platelets at high shear rates (1500 s^−1^ and 5000 s^−1^) (Fig. 6) indicate that the diminished platelet stable adhesion and irreversible aggregation impair the stabilization and growth of thrombi under higher flow and thus may prevent the complete occlusion of the stenotic medium-sized arteries in contrast to an adequate hemostatic situation that may be achieved in normal small vessels.

Integrins are αβ heterodimers and the β3 subunit forms αIIbβ3 and αvβ3 complexes; the former is expressed exclusively in megakaryocyte/platelet lineage and the latter is however expressed by multiple cell types [43]. Integrin αvβ3 is also present in platelet, but only a few hundred copies in contrast to approximately 80,000 αIIbβ3 per platelet. Disruption of the Src/β3 interaction by the RGT peptide interferes with signal transduction through integrin αIIbβ3 in platelets and may also affect that through integrin αvβ3 in platelets and other cells such as melanoma cells or osteoclasts. The influence of the RGT-containing peptide on platelet function may be contributed by the global action of the peptide on the platelet β3 integrins while, as a commonly accepted concept, αIIbβ3 but not αvβ3 is crucial to platelet function owing to the overwhelming expression superiority. It would also be interesting to look at the effect of blocking the Src/β3 interaction in integrin αvβ3-expressing cells on tumorogenesis or bone resorption. That would be the goals for the future work.

## Conclusions

The present study reveals novel insights into the molecular mechanisms by which c-Src regulates integrin αIIbβ3 signaling and provides first evidence in human platelets that the RGT peptide or its derivatives inhibit outside-in signaling by dissociating the Src/β3 interaction independent of membrane anchorage. This work lays the foundation for an in vivo validation of the effect of RGT peptide by using membrane-permeable small molecules derived from structure-based design and allows us to anticipate that intercellular delivery of the RGT peptide analogues may have the potential to be developed into a novel antithrombotic strategy where only the Src/β3 interaction is specifically disrupted.

## Materials and methods

### Antibodies and reagents

Antibody SZ-21 specific for integrin β3 was provided by C. Ruan (Jiangsu Institute of Hematology, Suzhou, China) and 7E3 against integrin αIIbβ3 from J. Liu (Shanghai Jiao Tong University School of Medicine, Shanghai, China). Anti-Csk and anti-human IL-2 receptor α antibodies were purchased from Santa Cruz Biotechnology and R&D systems, respectively. Anti-β3-pY^747^ and anti-β3-pY^759^ antibodies were purchased from Abcam, and anti-Src, anti-Src-pY^416^, and anti-Src-pY^527^ antibodies were from Cell Signaling. FITC-anti-CD62P antibody was a product of Beckman Coulter. Purified active c-Src and the Src kinase assay kit were from Upstate Biotechnology. Collagen type I was from Chrono-log. D-Phe-Pro-Arg chloromethylketone (PPACK) was from Enzo Life Sciences. All other reagents were obtained from Sigma-Aldrich.

### Peptide synthesis

The cell membrane-permeable and reduction-sensitive fluorescent peptide (FAM)myr-KACKK ~ (TAMRA)CRGT and the RGT, CRGT, CGRT, myr-AC ~ CRGT, myr-AC ~ CGRT peptides as well as the integrin β3 cytoplasmic peptides (Additional file 6: Table S1) were all synthesized by the Chinese Peptide Company.

### The cDNA constructs and cell lines

The cDNA encoding chimeric Tac-β3 was constructed into the CMV-IL2R vector as previously described [44]. The cDNA encoding Tac-β3-ΔRGT was created using a site-directed mutagenesis kit (Stratagene). Full-length human c-Src cDNA was a gift from X. Gong (Zhejiang University, Hangzhou, China). The c-Src Y^527^F mutant was generated by site-directed mutagenesis, and a Flag tag was added at the 3′ end of the c-Src Y^527^F sequence. CHO cells were cultured as previously described [45].

### Detection of platelet uptake of the reduction-sensitive fluorescent peptides

Washed platelets were treated with 250 μM of (FAM)myr-KACKK ~ (TAMRA)CRGT and the time course fluorescence measurements (*λ*ex = 495 nm, *λ*em = 519 nm for FAM) were carried out using a TECAN Safire^2^ microplate reader at 37 °C. To determine the efficiency of CRGT release in cytoplasm, platelets were incubated with 250 μM of (FAM)myr-KACKK ~ (TAMRA)CRGT for different time periods, washed twice with CGS, and resuspended in HEPES-Tyrode’s buffer. The samples were measured at 495 or 546 nm as excitation and 519 nm (FAM) or 576 nm (TAMRA) as emission wavelength. The ratio of FAM to TAMRA fluorescence quantum was calculated. As control of a drastic reduction, 250 μM of (FAM)myr-KACKK ~ (TAMRA)CRGT was treated with 0.3 M of β-ME. To further determine the distribution of the CRGT peptide, the samples were examined with a Zeiss LSM 510 laser confocal microscope using a × 100 oil immersion objective as previously described [25].

### Phosphorylation of the tyrosines at position 747 and 759 of the integrin β3 tail by active c-Src

First, the Y^747^ and Y^759^ phosphorylation of the β3 tail was estimated by MALDI-TOF MS. The purified β3 cytoplasmic peptides, with wild type or Y^759^F, Y^747^F, Y^747, 759^ F, ΔRGT Y^747^F, ΔRGT Y^759^F, and ΔRGT mutant sequences were dissolved in Src kinase reaction buffer. The peptides (at a final concentration of 20 μM) in 10 μL of Src kinase reaction buffer were mixed with purified active c-Src (1 unit) and incubated at 30 °C for 3 h with the manganese/ATP cocktail. The samples were subjected to MALDI-TOF mass spectrometry analysis after desalted on a C18 tip and eluted with acetonitrile, water, and trifluoroacetic acid (90/10/0.1, by volume). The Y^747^ and Y^759^ phosphorylation of β3 was further determined by a [γ-^32^P] incorporation assay [27]. Briefly, The aforementioned β3 cytoplasmic peptides or a control substrate peptide KVEKIGEGTYGVVYK were incubated with purified active c-Src in Src kinase reaction buffer containing 5 μCi of [γ-^32^P]ATP and 10 μM of ATP at 30 °C overnight. Reaction mixtures were transferred to P81 phosphocellulose membranes and washed with 0.75 % phosphoric acid. These membranes were rinsed briefly in acetone, air dried, and transferred into scintillation vials. The [γ-^32^P]ATP incorporation of the peptides were counted using a Beckman LS6500 liquid scintillation counter (Beckman, Fullerton, CA). We also measured β3 Y^747^ and Y^759^ phosphorylation by active c-Src in CHO cell models. Tac-β3 or Tac-β3-ΔRGT was transfected or co-transfected together with c-Src Y^527^F at a ratio of 1:1 into CHO cells using the Lipofectamine 2000 kit (Invitrogen). Twenty-four hours later, the cells were lysed at 4 °C in ice-cold RIPA buffer (1 % NP-40, 150 mM NaCl, 50 mM Tris, pH 7.4, 1 mM EDTA, 0.25 % sodium deoxycholate, 1 mM sodium vanadate and 1 mM PMSF) and clarified by centrifugation at 12,000 rpm for 15 min and then were analyzed by Western blot.

### Platelet preparation, platelet spreading on immobilized fibrinogen, clot retraction assay, platelet aggregation, soluble fibrinogen binding, and P-selectin expression

Whole blood was drawn by venipuncture from healthy volunteers with informed consent who had not taken any medication within 2 weeks anticoagulated with 1/10 volume of 3.8 % (*w*/*v*) trisodium citrate. Platelet-rich plasma (PRP) was obtained by a centrifugation at 200 × *g* for 20 min. For preparation of washed platelets, the PRP was acidified by adding 1/7 volume of acid citrate dextrose (ACD; 85 mM trisodium citrate, 83 mM dextrose and 21 mM citric acid) and platelets were collected by a centrifugation at 1500 × *g* for 20 min. The platelet sediments were washed twice with CGS (120 mM NaCl, 13 mM trisodium citrate, 30 mM glucose, pH 6.5), and were finally resuspended at required concentrations in HEPES-Tyrode’s buffer (137 mM NaCl, 2 mM KCl, 12 mM NaHCO_3_, 0.3 mM NaH_2_PO_4_, 5.5 mM glucose, 1 mM CaCl_2_, 1 mM MgCl_2_, 5 mM HEPES, and 0.1 % BSA, pH 7.4). The platelet suspensions were left at room temperature for 60 min prior to being used in experiments. Platelet spreading on immobilized fibrinogen was assayed as previously described [15]. The 6-well microtiter plates were coated with human fibrinogen (20 μg/mL) in 0.1 M of NaHCO_3_ (pH 8.3) at 4 °C overnight. Washed platelets, resuspended at a final concentration of 1 × 10^8^/mL in HEPES-Tyrode’s buffer, were preincubated with peptides for 60 min and were then added into the fibrinogen-coated wells and incubated at 37 °C for 45 min. The platelets were collected and lysed for 30 min at 4 °C in ice-cold RIPA buffer containing the protease inhibitor mixture. After a centrifugation of the platelet lysates at 12,000 rpm for 15 min, the supernatants were added with an equal volume of 2 × SDS Laemmli sample buffer. Proteins were separated by SDS-PAGE using 12 % gels, transferred to PVDF membrane (Amersham), and immunoblotted with the antibodies specific for human c-Src, Src phospho-tyrosine^527^, Src phospho-tyrosine^416^, and actin. Clot retraction studies, platelet aggregation, soluble fibrinogen binding assay, and detection of P-selectin expression were performed as previously described [15].

### RhoA activation

Clot retraction was terminated at 60 min by the addition of lysis buffer. RhoA activation of platelets in clots was detected using an Active Rho Pull-Down and Detection Kit (Pierce Biotechnology) according to the published procedure with minor modifications [16].

### In vitro assays for c-Src activity

Reactions were carried out using a Src kinase assay kit (Upstate Biotechnology). Briefly, 150 μM of substrate peptide KVEKIGEGTYGVVYK was mixed with purified active c-Src in the presence or absence of CRGT, CGRT, or PP2 in Src kinase reaction buffer containing 5 μCi of [γ-^32^P]ATP and 10 μM of ATP and incubated at 30 °C overnight. [γ-^32^P]ATP incorporation was counted using a Beckman LS6500 liquid scintillation counter.

### Co-immunoprecipitation and Western blot analysis

Preparation of platelet lysates or cell lysates, immunoprecipitation, SDS-PAGE, and immunoblotting were performed according to the procedures published elsewhere [15]. In brief, the peptide-pretreated platelets, CHO cells co-expressing Tac-β3 and c-Src Y^527^F, or Tac-β3-ΔRGT and c-Src Y^527^F, or peptide-pretreated CHO cells co-expressing Tac-β3 and c-Src Y^527^F were lysed for 30 min at 4 °C in ice-cold RIPA buffer containing the protease inhibitor mixture. Protein concentrations were determined using the bicinchoninic acid protein assay kit (Pierce Chemical). The lysates were centrifuged for 5 min at 4 °C at 12,000 rpm, and then, 100 μL aliquots of the supernatants containing equal amounts of protein (ranging from 400 to 500 μg between experiments) were immunoprecipitated by the indicated antibodies for 2 h at 4 °C with gentle agitation and were further incubated with protein G plus-agarose beads overnight at 4 °C. After washing three times with RIPA buffer, the immunoprecipitates were eluted by boiling the beads in Laemmli sample buffer and were electrophoresed with 8 % or 12 % SDS-PAGE, transferred onto PVDF membrane. Proteins were detected using the indicated primary antibodies and HRP-conjugated secondary antibodies. Immunoreactive bands were detected by ECL with the reaction time ranging from 30 s to 5 min. The signal intensity of the respective bands was measured by the NIH Image J software.

### Ex vivo flow-based platelet adhesion and aggregation assays

The experiments were performed as described [46]. Bioflux 48 well plates (Fluxionbio) were coated with 20 μg/ml collagen at 4 °C overnight. Blood was drawn into PPACK (at a final concentration of 150 μM), and calcein AM (4 mM, at 37 °C for 30 min) was used to label platelets in whole blood. The blood was then incubated with peptides for 30 min at 37 °C and was perfused through the micro-chambers at wall shear rates of 125, 500, 1500 or 5000 s^−1^ for 4 min. Adherent platelet aggregates were monitored using an inverted fluorescent microscope with a × 20 long-working-distance objective and CCD camera (Nikon eclipse Ti-s).

### Statistics

Differences between groups were assessed using the Student’s *t* test. When three or more groups were compared, we used ANOVA (SPSS software, version 18). Data are presented as mean and SD, unless otherwise specified.

## Additional files

Additional file 1: Figure S1. Active c-Src directly phosphorylated the Y^747^ and Y^759^ residues of β3. (A) The amino acid sequences for the wild type or differently mutated integrin β3 cytoplasmic peptides. (B) MALDI-TOF mass spectrometry results of the integrin β3 cytoplasmic peptides (wild type or differently mutated) incubated with or without active c-Src. Black arrow shows the new peak which represents the addition of a phosphate group to the tyrosine of the peptides, and blank arrow shows that of two phosphate groups to two tyrosines. (C) The Src-induced [γ-^32^P]ATP incorporation assays were performed in the presence of a Src substrate peptide or different integrin β3 cytoplasmic peptides. The [γ-^32^P]ATP incorporation was observed in the peptide with tyrosine residue(s), but not in the Y^747^F and Y^759^F double mutated peptide. The Src inhibitor PP2 (20 μM) prevented the [γ-^32^P]ATP incorporation. Results are presented as mean and SD of three independent experiments. Additional file 2: Figure S2. Myr-AC ~ CRGT peptide impaired integrin αIIbβ3-mediated outside-in signaling. (A) The peptide-pretreated platelets were allowed to adhere on fibrinogen-coated coverslips for 45 min. Data shown are representative pictures from one of three experiments with similar results. Scale bar is 20 μm. (B) The percentage of the surface areas covered by spreading platelets. (C) The adherent platelets after washing were quantified by a PNPP assay. Washed platelets were incubated with different peptides as indicated. (D) After incubation with myr-AC ~ CRGT or control peptide and deposited on immobilized fibrinogen for 45 min, the platelets were lysed and analyzed for the activation of c-Src by Western blot. (E) Fibrin clot formation was initiated by adding 1 U/mL thrombin in the presence of 2 mg/mL of human fibrinogen. The clots were photographed at different time points. (F) The percentage of the clot size was generated by calculating the ratio of the surface area of the retracted clots versus that of the initial clots. The data are presented as the mean and SD of three independent experiments. (G) The fibrin clots containing platelets were lysed and analysed for c-Src and RhoA activation by Western blot. (H) After an incubation with DMSO (a), 250 μM of myr-AC ~ CGRT (b), myr-AC ~ CRGT at concentrations of 62.5 μM (c), 125 μM (d) or 250 μM (e), platelet aggregation was induced by ADP, thrombin, ristocetin, or collagen. (I) Surface expression of P-selectin on untreated or peptide-pretreated platelets stimulated with thrombin (0.1 U/mL). (J) The fluorescence intensity calculated from the data in panel I is presented as the mean and SD of three independent experiments. Additional file 3: Figure S3. Myr-AC ~ CRGT peptide did not affect the integrin αIIbβ3-mediated inside-out signaling. (A) Effect of myr-AC ~ CRGT on soluble fibrinogen binding to platelets. Platelets were preincubated with different peptides or their vehicles, and binding of Alexa Fluor 488-conjugated fibrinogen (100 μg/mL) to platelets was measured by flow cytometry after the addition of 20 μM ADP. (B) The fluorescence intensity calculated from the data in panel A is presented as the mean and SD of three independent experiments. Additional file 4: Figure S4. Myr-AC ~ CRGT dose-dependently inhibited thrombus formation of human platelets at a shear rate of 1500 s^−1^. Calcein AM-labeled whole blood was preincubated as indicated and then perfused through a collagen-coated surface at a wall shear rate of 1500 s^−1^ for 4 min. The thrombus formation was observed and imaged under an inverted fluorescent microscope with a × 20 long-working-distance objective. (A) The representative images showed platelet thrombi (scale bar is 100 μm). (B) The quantitative data were acquired as platelet-integrated total fluorescence intensity. (C) The percentage of surface coverage. (D) The mean fluorescence intensity calibrated by surface coverage. Results are the mean and SD from three independent experiments. **P* < 0.01. Additional file 5: Figure S5. Myr-AC ~ CRGT peptide did not cause cell lysis. (A) Release of hemoglobin from human erythrocytes induced by various concentrations of myr-AC ~ CRGT or the scrambled myr-AC ~ CGRT. The extent of the hemoglobin release into the supernatant was measured at 405 nm. Triton X-100- or DMSO-treated erythrocytes served as positive or negative controls, respectively. (B) Release of phosphatase activity from nonaggregated platelets. Platelets were incubated with peptide as indicated at 37 °C for 30 min in the absence of stirring. The phosphatase activity was separately measured in platelet-free supernatant (black bar) or platelet pellets (white bar) by using p-nitrophenyl phosphate as a chromogenic substrate. The mean and SD were obtained from three independent experiments. Additional file 6: Table S1. The integrin β3 cytoplasmic peptides (wild type or differently mutated).

## Abbreviations

GPIb: Glycoprotein Ib
vWF: von Willebrand factor
GPVI: Glycoprotein VI
ADP: Adenosine diphosphate
PAR: Protease-activated receptor
SH3: Src homology 3
SH2: Src homology 2
β-ME: β-mercaptoethanol
ATP: Adenosine triphosphate
CHO: Chinese hamster ovary

## References

[1] Ruggeri ZM (2002). Platelets in atherothrombosis. Nat Med.

[2] Nieswandt B, Watson SP (2003). Platelet-collagen interaction: is GPVI the central receptor?. Blood.

[3] Takizawa H, Nishimura S, Takayama N, Oda A, Nishikii H, Morita Y (2010). Lnk regulates integrin alphaIIbbeta3 outside-in signaling in mouse platelets, leading to stabilization of thrombus development in vivo. J Clin Invest.

[4] Moser M, Legate KR, Zent R, Fassler R (2009). The tail of integrins, talin, and kindlins. Science.

[5] Arias-Salgado EG, Lizano S, Shattil SJ, Ginsberg MH (2005). Specification of the direction of adhesive signaling by the integrin beta cytoplasmic domain. J Biol Chem.

[6] Arias-Salgado EG, Lizano S, Sarkar S, Brugge JS, Ginsberg MH, Shattil SJ (2003). Src kinase activation by direct interaction with the integrin beta cytoplasmic domain. Proc Natl Acad Sci U S A.

[7] Law DA, DeGuzman FR, Heiser P, Ministri-Madrid K, Killeen N, Phillips DR (1999). Integrin cytoplasmic tyrosine motif is required for outside-in alphaIIbbeta3 signalling and platelet function. Nature.

[8] Nanda N, Andre P, Bao M, Clauser K, Deguzman F, Howie D (2005). Platelet aggregation induces platelet aggregate stability via SLAM family receptor signaling. Blood.

[9] Goschnick MW, Lau LM, Wee JL, Liu YS, Hogarth PM, Robb LM (2006). Impaired “outside-in” integrin alphaIIbbeta3 signaling and thrombus stability in TSSC6-deficient mice. Blood.

[10] Angelillo-Scherrer A, de Frutos P, Aparicio C, Melis E, Savi P, Lupu F (2001). Deficiency or inhibition of Gas6 causes platelet dysfunction and protects mice against thrombosis. Nat Med.

[11] Stolla M, Stefanini L, Roden RC, Chavez M, Hirsch J, Greene T (2011). The kinetics of alphaIIbbeta3 activation determines the size and stability of thrombi in mice: implications for antiplatelet therapy. Blood.

[12] Konopatskaya O, Gilio K, Harper MT, Zhao Y, Cosemans JM, Karim ZA (2009). PKCalpha regulates platelet granule secretion and thrombus formation in mice. J Clin Invest.

[13] Naik MU, Nigam A, Manrai P, Millili P, Czymmek K, Sullivan M (2009). CIB1 deficiency results in impaired thrombosis: the potential role of CIB1 in outside-in signaling through integrin alpha IIb beta 3. J Thromb Haemost.

[14] Rossaint J, Vestweber D, Zarbock A (2013). GDF-15 prevents platelet integrin activation and thrombus formation. J Thromb Haemost.

[15] Su X, Mi J, Yan J, Flevaris P, Lu Y, Liu H (2008). RGT, a synthetic peptide corresponding to the integrin beta 3 cytoplasmic C-terminal sequence, selectively inhibits outside-in signaling in human platelets by disrupting the interaction of integrin alpha IIb beta 3 with Src kinase. Blood.

[16] Shen B, Zhao X, O’Brien KA, Stojanovic-Terpo A, Delaney MK, Kim K (2013). A directional switch of integrin signalling and a new anti-thrombotic strategy. Nature.

[17] Huveneers S, Danen EH (2010). The interaction of SRC kinase with beta3 integrin tails: a potential therapeutic target in thrombosis and cancer. Sci World J.

[18] Shattil SJ (2005). Integrins and Src: dynamic duo of adhesion signaling. Trends Cell Biol.

[19] Bennett JS (2008). Outside-in: peptide versus integrin. Blood.

[20] Moarefi I, LaFevre-Bernt M, Sicheri F, Huse M, Lee CH, Kuriyan J (1997). Activation of the Src-family tyrosine kinase Hck by SH3 domain displacement. Nature.

[21] Xiao R, Xi XD, Chen Z, Chen SJ, Meng G (2013). Structural framework of c-Src activation by integrin beta3. Blood.

[22] Prevost N, Woulfe DS, Jiang H, Stalker TJ, Marchese P, Ruggeri ZM (2005). Eph kinases and ephrins support thrombus growth and stability by regulating integrin outside-in signaling in platelets. Proc Natl Acad Sci U S A.

[23] Andre P, Prasad KS, Denis CV, He M, Papalia JM, Hynes RO (2002). CD40L stabilizes arterial thrombi by a beta3 integrin–dependent mechanism. Nat Med.

[24] Willenbrock F, Thomas DA, Amour A (2010). Kinetic analysis of the inhibition of matrix metalloproteinases: lessons from the study of tissue inhibitors of metalloproteinases. Methods Mol Biol.

[25] Ensenat-Waser R, Martin F, Barahona F, Vazquez J, Soria B, Reig JA (2002). Direct visualization by confocal fluorescent microscopy of the permeation of myristoylated peptides through the cell membrane. IUBMB Life.

[26] Licht T, Tsirulnikov L, Reuveni H, Yarnitzky T, Ben-Sasson SA (2003). Induction of pro-angiogenic signaling by a synthetic peptide derived from the second intracellular loop of S1P3 (EDG3). Blood.

[27] Mahabeleshwar GH, Feng W, Reddy K, Plow EF, Byzova TV (2007). Mechanisms of integrin-vascular endothelial growth factor receptor cross-activation in angiogenesis. Circ Res.

[28] Ruggeri ZM (1999). Structure and function of von Willebrand factor. Thromb Haemost.

[29] Farndale RW (2006). Collagen-induced platelet activation. Blood Cells Mol Dis.

[30] Shattil SJ, Kashiwagi H, Pampori N (1998). Integrin signaling: the platelet paradigm. Blood.

[31] Savage B, Almus-Jacobs F, Ruggeri ZM (1998). Specific synergy of multiple substrate-receptor interactions in platelet thrombus formation under flow. Cell.

[32] Savage B, Ginsberg MH, Ruggeri ZM (1999). Influence of fibrillar collagen structure on the mechanisms of platelet thrombus formation under flow. Blood.

[33] Eichholtz T, de Bont DB, de Widt J, Liskamp RM, Ploegh HL (1993). A myristoylated pseudosubstrate peptide, a novel protein kinase C inhibitor. J Biol Chem.

[34] Murray D, Ben-Tal N, Honig B, McLaughlin S (1997). Electrostatic interaction of myristoylated proteins with membranes: simple physics, complicated biology. Structure.

[35] Haramaki N, Ikeda H, Takajo Y, Katoh A, Kanaya S, Shintani S (2001). Long-term smoking causes nitroglycerin resistance in platelets by depletion of intraplatelet glutathione. Arterioscler Thromb Vasc Biol.

[36] Heemskerk JW, Sakariassen KS, Zwaginga JJ, Brass LF, Jackson SP, Farndale RW (2011). Collagen surfaces to measure thrombus formation under flow: possibilities for standardization. J Thromb Haemost.

[37] Stalker TJ, Traxler EA, Wu J, Wannemacher KM, Cermignano SL, Voronov R (2013). Hierarchical organization in the hemostatic response and its relationship to the platelet-signaling network. Blood.

[38] Tschopp TB, Weiss HJ, Baumgartner HR (1975). Interaction of thrombasthenic platelets with subendothelium: normal adhesion, absent aggregation. Experientia.

[39] Sakariassen KS, Nievelstein PF, Coller BS, Sixma JJ (1986). The role of platelet membrane glycoproteins Ib and IIb-IIIa in platelet adherence to human artery subendothelium. Br J Haematol.

[40] Kroll MH, Hellums JD, McIntire LV, Schafer AI, Moake JL (1996). Platelets and shear stress. Blood.

[41] Nesbitt WS, Westein E, Tovar-Lopez FJ, Tolouei E, Mitchell A, Fu J (2009). A shear gradient-dependent platelet aggregation mechanism drives thrombus formation. Nat Med.

[42] Bark DL, Ku DN (2010). Wall shear over high degree stenoses pertinent to atherothrombosis. J Biomech.

[43] Desgrosellier JS, Barnes LA, Shields DJ, Huang M, Lau SK, Prevost N (2009). An integrin alpha(v)beta(3)-c-Src oncogenic unit promotes anchorage-independence and tumor progression. Nat Med.

[44] Chen YP, O’Toole TE, Shipley T, Forsyth J, LaFlamme SE, Yamada KM (1994). “Inside-out” signal transduction inhibited by isolated integrin cytoplasmic domains. J Biol Chem.

[45] Xi X, Bodnar RJ, Li Z, Lam SC, Du X (2003). Critical roles for the COOH-terminal NITY and RGT sequences of the integrin beta3 cytoplasmic domain in inside-out and outside-in signaling. J Cell Biol.

[46] Valera MC, Gratacap MP, Gourdy P, Lenfant F, Cabou C, Toutain CE (2012). Chronic estradiol treatment reduces platelet responses and protects mice from thromboembolism through the hematopoietic estrogen receptor alpha. Blood.

