# Supplementary figures and images for: Evaluation of targeting c-Src by the RGT-containing peptide as a novel antithrombotic strategy
Source: J Hematol Oncol. 2015 May 30;8:62. doi: 10.1186/s13045-015-0159-8 (PMC4459659; doi:10.1186/s13045-015-0159-8)

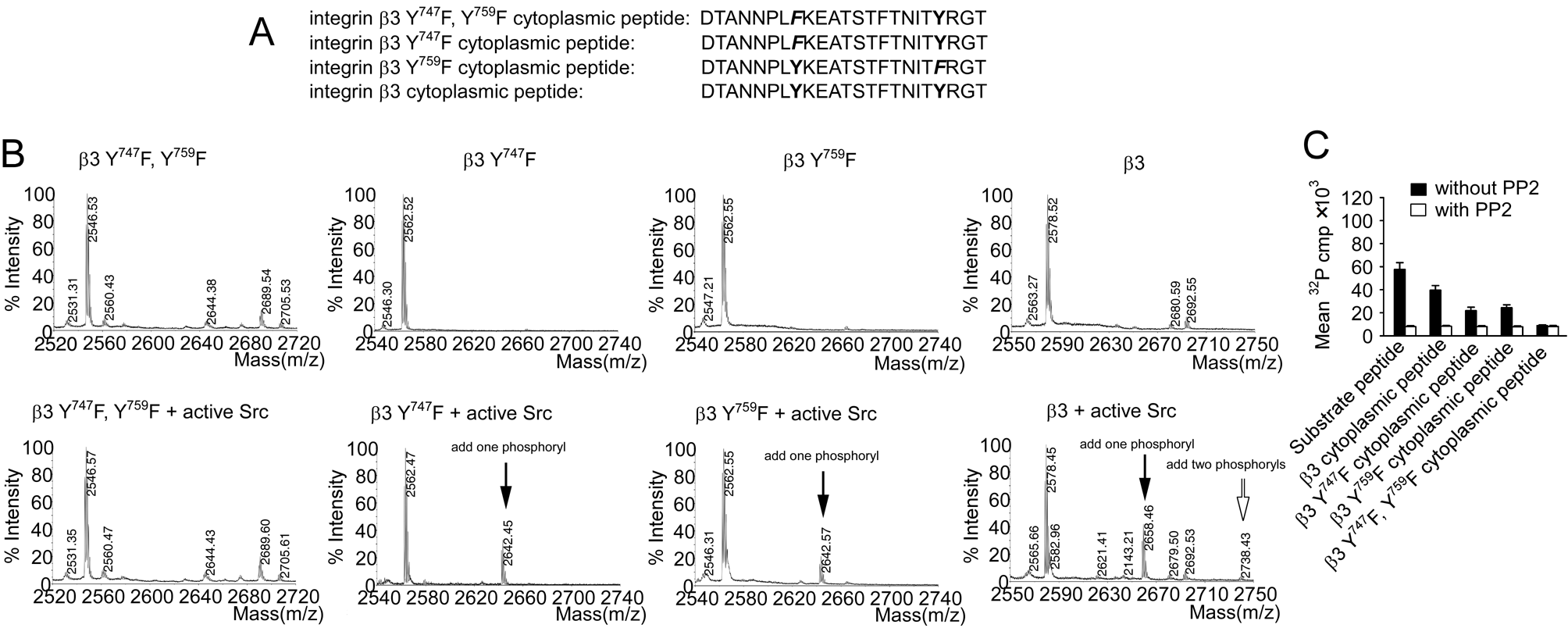

Supplement: Additional file 1: Figure S1. — Active c-Src directly phosphorylated the Y747 and Y759 residues of β3. (A) The amino acid sequences for the wild type or differently mutated integrin β3 cytoplasmic peptides. (B) MALDI-TOF mass spectrometry results of the integrin β3 cytoplasmic peptides (wild type or differently mutated) incubated with or without active c-Src. Black arrow shows the new peak which represents the addition of a phosphate group to the tyrosine of the peptides, and blank arrow shows that of two phosphate groups to two tyrosines. (C) The Src-induced [γ-32P]ATP incorporation assays were performed in the presence of a Src substrate peptide or different integrin β3 cytoplasmic peptides. The [γ-32P]ATP incorporation was observed in the peptide with tyrosine residue(s), but not in the Y747F and Y759F double mutated peptide. The Src inhibitor PP2 (20 μM) prevented the [γ-32P]ATP incorporation. Results are presented as mean and SD of three independent experiments. [file 13045_2015_159_MOESM1_ESM.tif]

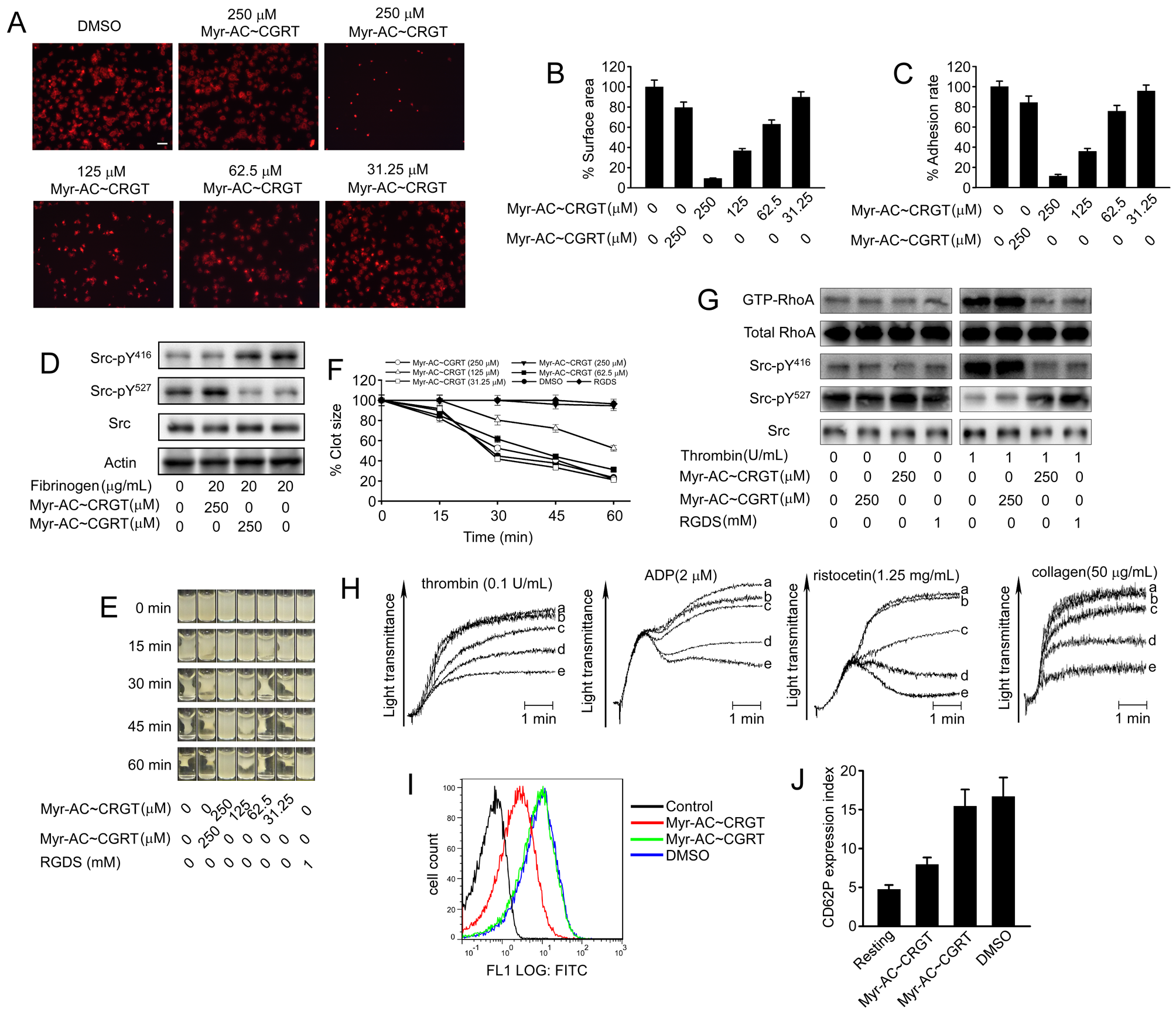

Supplement: Additional file 2: Figure S2. — Myr-AC ~ CRGT peptide impaired integrin αIIbβ3-mediated outside-in signaling. (A) The peptide-pretreated platelets were allowed to adhere on fibrinogen-coated coverslips for 45 min. Data shown are representative pictures from one of three experiments with similar results. Scale bar is 20 μm. (B) The percentage of the surface areas covered by spreading platelets. (C) The adherent platelets after washing were quantified by a PNPP assay. Washed platelets were incubated with different peptides as indicated. (D) After incubation with myr-AC ~ CRGT or control peptide and deposited on immobilized fibrinogen for 45 min, the platelets were lysed and analyzed for the activation of c-Src by Western blot. (E) Fibrin clot formation was initiated by adding 1 U/mL thrombin in the presence of 2 mg/mL of human fibrinogen. The clots were photographed at different time points. (F) The percentage of the clot size was generated by calculating the ratio of the surface area of the retracted clots versus that of the initial clots. The data are presented as the mean and SD of three independent experiments. (G) The fibrin clots containing platelets were lysed and analysed for c-Src and RhoA activation by Western blot. (H) After an incubation with DMSO (a), 250 μM of myr-AC ~ CGRT (b), myr-AC ~ CRGT at concentrations of 62.5 μM (c), 125 μM (d) or 250 μM (e), platelet aggregation was induced by ADP, thrombin, ristocetin, or collagen. (I) Surface expression of P-selectin on untreated or peptide-pretreated platelets stimulated with thrombin (0.1 U/mL). (J) The fluorescence intensity calculated from the data in panel I is presented as the mean and SD of three independent experiments. [file 13045_2015_159_MOESM2_ESM.tif]

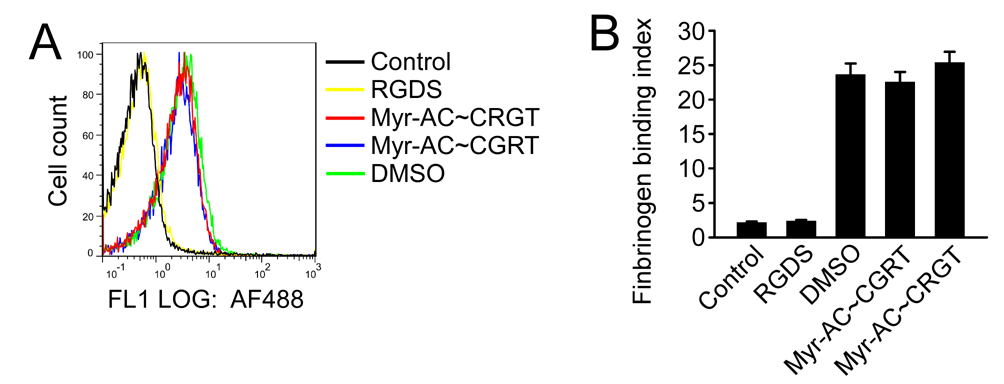

Supplement: Additional file 3: Figure S3. — Myr-AC ~ CRGT peptide did not affect the integrin αIIbβ3-mediated inside-out signaling. (A) Effect of myr-AC ~ CRGT on soluble fibrinogen binding to platelets. Platelets were preincubated with different peptides or their vehicles, and binding of Alexa Fluor 488-conjugated fibrinogen (100 μg/mL) to platelets was measured by flow cytometry after the addition of 20 μM ADP. (B) The fluorescence intensity calculated from the data in panel A is presented as the mean and SD of three independent experiments. [file 13045_2015_159_MOESM3_ESM.tif]

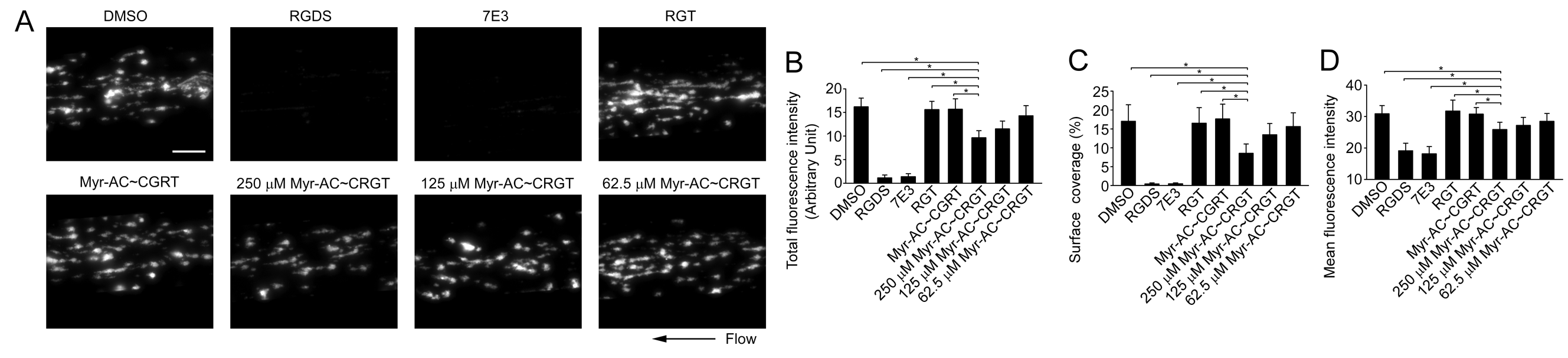

Supplement: Additional file 4: Figure S4. — Myr-AC ~ CRGT dose-dependently inhibited thrombus formation of human platelets at a shear rate of 1500 s−1. Calcein AM-labeled whole blood was preincubated as indicated and then perfused through a collagen-coated surface at a wall shear rate of 1500 s−1 for 4 min. The thrombus formation was observed and imaged under an inverted fluorescent microscope with a × 20 long-working-distance objective. (A) The representative images showed platelet thrombi (scale bar is 100 μm). (B) The quantitative data were acquired as platelet-integrated total fluorescence intensity. (C) The percentage of surface coverage. (D) The mean fluorescence intensity calibrated by surface coverage. Results are the mean and SD from three independent experiments. *P < 0.01. [file 13045_2015_159_MOESM4_ESM.tif]

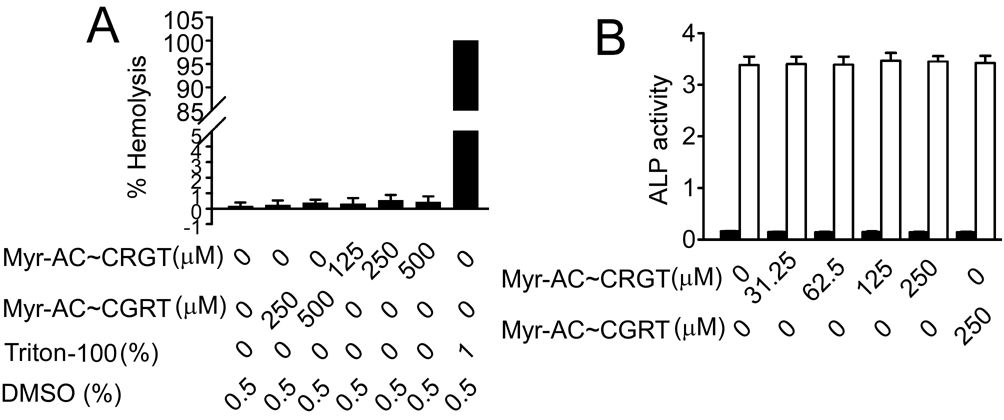

Supplement: Additional file 5: Figure S5. — Myr-AC ~ CRGT peptide did not cause cell lysis. (A) Release of hemoglobin from human erythrocytes induced by various concentrations of myr-AC ~ CRGT or the scrambled myr-AC ~ CGRT. The extent of the hemoglobin release into the supernatant was measured at 405 nm. Triton X-100- or DMSO-treated erythrocytes served as positive or negative controls, respectively. (B) Release of phosphatase activity from nonaggregated platelets. Platelets were incubated with peptide as indicated at 37 °C for 30 min in the absence of stirring. The phosphatase activity was separately measured in platelet-free supernatant (black bar) or platelet pellets (white bar) by using p-nitrophenyl phosphate as a chromogenic substrate. The mean and SD were obtained from three independent experiments. [file 13045_2015_159_MOESM5_ESM.tif]
